# INdependenT prEscribinG in community phaRmAcy; whaT works for whom, why and in what circumstancEs (INTEGRATE): Realist review study protocol

**DOI:** 10.3310/nihropenres.13766.1

**Published:** 2024-11-18

**Authors:** Ola Amr Abdelfatah, Andrea Hilton, Ellen Schafheutle, Geof Wong, Keith Holden, Lesley Scott, Nia Roberts, Nick Haddington, Tony Kelly, Vivienne Hibberd, Andrew Sturrock, Ian Maidment

**Affiliations:** 1 Aston Pharmacy School, Aston University Aston Pharmacy School, Birmingham, England, B47ET, UK; 2 Paramedical, Perioperative and Advanced Practice; Faculty of Health Sciences, University of Hull Department of Paramedical Perioperative and Advanced Practice, Hull, England, HU6 7RX, UK; 3 Centre for Pharmacy Workforce Studies, The University of Manchester Division of Pharmacy and Optometry, Manchester, England, M13 9PL, UK; 4 Nuffield Department of Primary Care Health Sciences, University of Oxford, Oxford, England, OX2 6GG, UK; 5 School of Pharmacy and Pharmaceutical Sciences, University of Sunderland, Sunderland, England, SR1 3SD, UK; 6 Bodleian Health Care Libraries, University of Oxford, Oxford, England, OX3 9DU, UK; 7 Workforce Training and Education Directorate, South West, NHS England, Bristol, England, BS1 6AG, UK; 8 NHS Birmingham and Solihull STP, Solihull, England, B1 1TT, UK; 9 NHS Education for Scotland, Medical Directorate, NHS Scotland, Edinbrugh, Scotland, EH39DN, UK

**Keywords:** Independent Prescribing, Community Pharmacist, Medication, Realist Review

## Abstract

**Introduction:**

The last decades have witnessed a series of initiatives in the United Kingdom (UK) to enhance patient access to quality care including access to medicine without compromising patient’s safety. Pharmacist independent prescribing is one of the initiatives introduced in 2006 with the intention of making more effective use of the skills and competencies of health professionals. Community pharmacy has a key role in the NHS long-term plan since pharmacies offer convenient and accessible sources of healthcare advice for the public. This role is more evident with the introduction of prescribing for all qualified pharmacists at the point of registration starting 2026. This realist review aims to explore how does independent prescribing in community pharmacy works, for whom, in what circumstances and how.

**Method and analysis:**

Realist research seeks to explore and explain complex social interventions by utilising programme theories providing causal explanations of outcomes in terms of context-mechanism-outcome configurations.

INTEGRATE will progress through six stages. In the first stage, we will partner with Patient, Public, Involvement and Engagement Group (PPIE) and Practitioner Stakeholder Group (SG), to further scrutinise the review’s focus. In stage 2, we will develop initial programme theories for what makes independent prescribing effective in community pharmacy, for whom, in what circumstances and how. In the third stage, we will conduct literature searches to gather secondary data that will help refine our initial programme theories.

In stage 4, we will select and appraise identified articles by screening titles, abstracts and full texts against inclusion and exclusion criteria. In stage 5, we will extract, document and code relevant data, followed by realist analysis with contributions from the PPIE and SG. Stage 6 focuses on refining programme theories and identifying key mechanisms that lead to desired outcomes.

PROSPERO registration: CRD42023468451

## Plain Language Summary

Community pharmacies can be found in practically all communities, and an increasing number of people are visiting pharmacies for care. Making better use of community pharmacists is a key part of the UK NHS Long-Term Plan ( Alderwick & Dixon, 2019). Pharmacists have been able to qualify as prescribers (meaning they can prescribe medicines without the need to involve a doctor) since 2006, after obtaining an extra qualification that they can pursue after two years of pharmacy working experience. In their trend data report, the General Pharmaceutical Council (GPhC) reported a total of 18,720 pharmacist independent prescribers on 30 April 2024, which represent 29.1% of all registered pharmacists. Starting 2026 onwards, all newly qualified pharmacists will be independent prescribers when they start working as pharmacists.

We need to better understand what opportunities and challenges there might be so that the NHS and the public get the most benefit from these changes. To do this we will undertake a review of what has previously been written (called a literature review) about community pharmacists as independent prescribers, to find out what works, for whom, why, and when. We will also look at what has been written about independent prescribing in other settings.

We are a team of healthcare professionals, researchers, and patients. We will make recommendations on how best to develop services.

## Introduction

Over the last 20 years a substantial change has been seen in the responsibilities and functions of all health professionals. The National Health Service (NHS) in the UK is currently undergoing unprecedented work pressures due to increasing patient demand, combined with staffing problems such as decreasing number of general practitioners (GPs), low morale and lower levels of funding available ( Paloumpi *et al.*, 2024). Consequently, the NHS is facing a so-called ‘workforce crisis’ ( Hindi *et al.*, 2019). General practice services, often the first point of contact, are under particular pressure ( Paloumpi *et al.*, 2023). Meanwhile, an ‘untapped’ and at times underutilised primary care workforce is community pharmacists. Community pharmacies offer convenient and accessible sources of healthcare advice for the public, particularly in areas of high deprivation ( Todd *et al.*, 2015). With independent prescribing qualifications and skills, pharmacists are able to manage and treat patients within community pharmacies, alleviating pressures on GPs and allowing GPs to treat more complex cases ( Alshakmobarak *et al.*, 2024).

Pharmacists have extensive knowledge of medicines, however without additional qualifications they are not currently able to prescribe. Pharmacists in the UK have been able to become independent prescribers since 2006 ( Mantzourani *et al.*, 2023), however few pharmacists are active prescribers, particularly in community pharmacy ( NHS, 2022). Making better use of community pharmacy is a key part of the NHS Long Term Plan ( Alderwick & Dixon, 2019). Community pharmacies are increasingly being accessed by patients for their healthcare needs. A recent audit found that a quarter of a million of the consultations that take place weekly in English community pharmacies are due to patients being unable to access other areas of the healthcare system ( Brown, 2022). Enabling this highly trained and skilled workforce to work at the ‘top of their scope of practice’ – especially when it comes to prescribing – is one potential solution to the workforce crisis and the challenges of patient access ( Mantzourani *et al.*, 2023).

There are no widespread, or nationally commissioned services in community pharmacy in England, which require prescribing. However, new regulator/GPhC Standards for the Education and Training of Pharmacists will see all pharmacy graduates from 2026 hold prescribing status on registration) ( GPhC, 2022). Additionally, several pathways and initiatives are set up to promote obtaining prescribing qualifications for already registered pharmacists such as the NHS Additional Role Reimbursement Scheme (ARRS), which provides support with funding training and access to supervision ( Bramwell *et al.*, 2024), and NHS England training offers for pharmacists ( NHS, 2024), which offer fully-funded clinical examination skills training for community pharmacists as well as independent prescribing training. 

Integrating prescribing as part of pharmacist educational reforms could enable pharmacists to better fulfil their roles as medicines experts and expand their scope of practice ( GPhC, 2022). However, the limited active prescribing, as reported by the community pharmacy workforce survey ( 2023), by existing qualified community pharmacists indicates that barriers exist that we do not fully understand ( Edwards *et al.*, 2022). This project seeks to address this issue now and is timely as it can produce the necessary evidence base as the first cohort of prescriber-ready pharmacists will join the workforce in the summer of 2026. The evidence and recommendations produced by this research will be key to influencing policy and service development and will be used to support the development of new services in pharmacies, embedded in the integrated care systems (ICS) that meet the needs of patients and realise pharmacists’ potential as prescribers. ICS refers to the partnerships that brings health leading organisations together to work on improving public health and well-being. Its primary goals are to shift health care out of hospital settings into community, combine health and social care services and address health inequalities ( Charles *et al.*, 2018). Thus, independent prescribing services in community pharmacy supports the broader goals of ICS to improve population access to care and enhance care coordination.

INTEGRATE is realist synthesis with extensive engagement with ‘experts-by-experience’ both patients and family carers and practitioners to make sense of the complexities and understand how independent prescribing in community pharmacy works, why it works, and in what circumstances. From this, recommendations will be developed to maximise implementation and utilisation to meet the needs of patients.

### Realist approach

Realist synthesis is theory-driven, meaning it is aimed at explaining how different contexts impact different mechanisms that lead to both expected and unexpected outcomes in programmes. They are set out to explore beyond intervention effectiveness and understanding why, how and in which context do interventions work ( Pawson, 2006). They explain causation using context, mechanism, outcome (CMO) configurations which are themselves organised into a programme theory. Realist methodology seeks to understand the deep generative mechanisms underlying intervention theories and uncover causal processes of complex social interventions ( Jagosh, 2019; Shearn *et al.*, 2017). The approach was chosen to inform the implementation of independent prescribing in community pharmacy, as it recognises that interventions used to implement prescribing may not always be universally successful and that outcomes can be dependent upon the context. The realist synthesis will be conducted following Realist and Meta-narrative Evidence Syntheses: Evolving Standards (RAMESES) guidelines for realist synthesis ( Wong *et al.*, 2015).

### Aims, objectives and review questions

This project will aim to understand how and why independent prescribing in community pharmacy works, or does not work, for which groups of people, and in which circumstances, through a realist synthesis of published evidence and grey literature. This will be achieved through synthesising collected evidence into one or more realist programme theories ( Pawson *et al.*, 2004). Based on the produced programme theories, recommendations will be co-produced with an expert stakeholder (SG) and PPIE groups that support widespread implementation of independent prescribing in community pharmacy ( Griffiths *et al.*, 2022). Initial review questions are detailed below.

***Overarching research question***

How does independent prescribing in community pharmacy work, why does it work, and in what circumstances?

**Realist synthesis sub-questions**

(1) What are the mechanisms, acting at a micro, meso and macro level, which lead to successful (or otherwise/ unsuccessful) implementation of independent prescribing in community pharmacy?
(2) What are the main contextual factors that influence whether the different mechanisms lead to successful or unsuccessful, implementation of independent prescribing in community pharmacy?
(3) What changes are needed to the existing and/or future service models to make safe and effective independent prescribing in community pharmacy more likely?
(4) What are the gaps in the evidence around implementation of independent prescribing in community pharmacy that future research needs to address?

## Methods

### Patient and Public Involvement

This study incorporates Patient and Public Involvement (PPI) throughout its design, execution, and dissemination phases, aligning with the NIHR’s commitment to embedding patient perspectives within health research, and underpinned by NIHR guidance ( NIHR, 2019) and UK Standards for Public Involvement ( NIHR, 2019b). Early in the project, we engaged patient and public representatives to identify key priorities and refine the study’s aims, ensuring that the research aligns with real-world needs and expectations. Our PPI group is made up of individuals from diverse backgrounds. This will ensure that the voices of underserved groups are heard, including people of different ethnic groups, gender, sexual orientation, people with disability and long-term conditions, people from rural and urban locations, and people living in areas of low socioeconomic status. Our PPI group is planned to meet regularly (up to 6 meetings) at key milestones throughout the project for their input into the development of patient facing study outputs, dissemination strategy and pathways to impact, this will include being offered co-authorship of outputs. Two members of the PPIE group will also be recruited to the project stakeholder group. More details on the involvement of PPI groups in each step of the process are given below.

### Realist synthesis process

**Figure 1.**
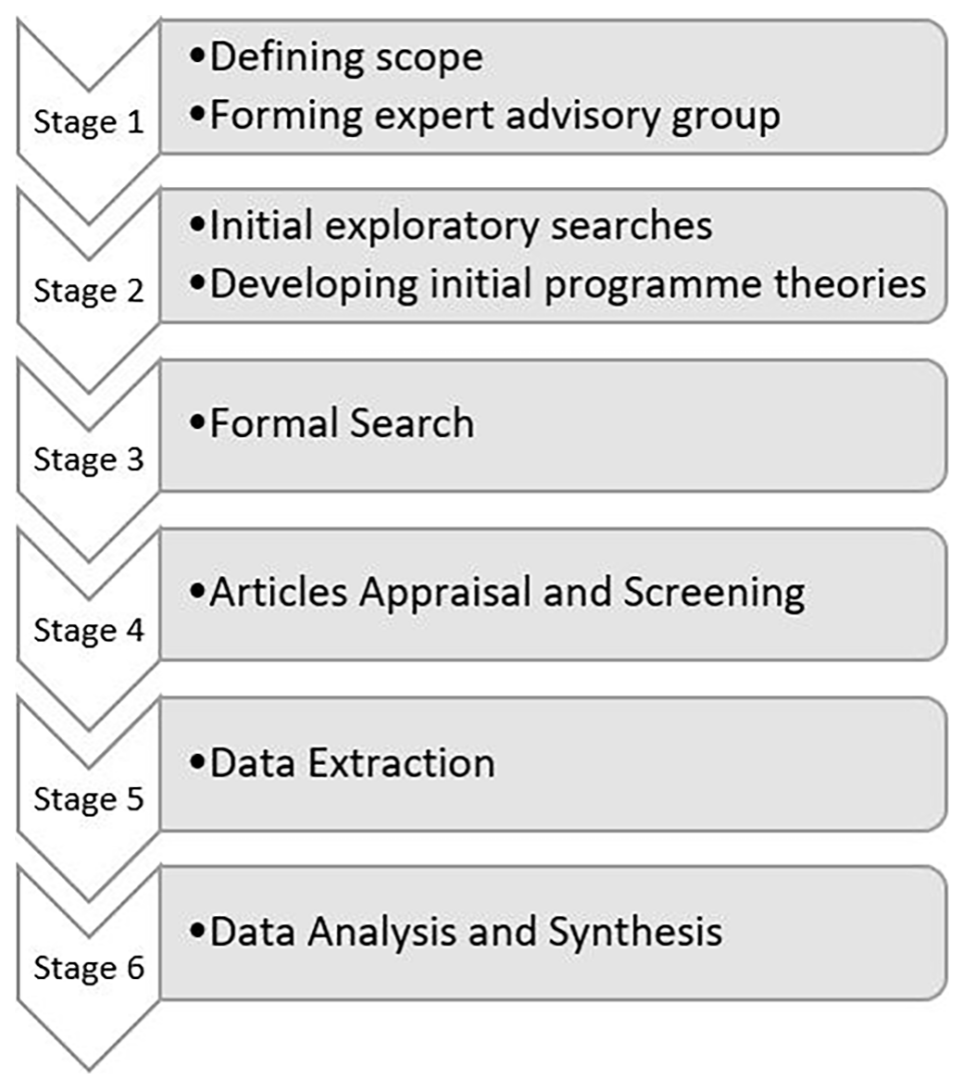
Review process.

The realist synthesis will follow an iterative six stage process in accordance with Pawson *et al.* (2005) methodology. The first stage starts with defining review scope, questions and forming expert advisory groups. The second stage includes locating existing theories via initial exploratory searches and developing initial programme theory with stakeholders. The third stage proceeds with a formal search for evidence, in which a search strategy will be developed by our information specialist to identify published research and grey literature. The team will then proceed with stage four, of evidence appraisal and screening against relevance criteria. Upon article selection, data extraction as stage five will commence and data for programme theory development will be coded in NVivo (version 14; QSR International, https://lumivero.com/products/nvivo/). Finally extracted data will be analysed and synthesised to draw conclusions in the final stage. This will be done through developing context-mechanism-outcome configurations and embedding them within programme theories that will undergo a process of continuous refinement with stakeholder input. Ethical approval is not required, because primary data will not be collected. This synthesis is registered on PROSPERO (ID: CRD42023468451). Figure 1 outlines the process of completing the review.

## Stage 1 – Define scope and form expert advisory group - PPI and stakeholder’s engagement

Two stakeholder’s advisory groups will be convened: a group consisting of patient and public involvement and engagement (PPIE) members and a practitioner stakeholder group (SG) comprising? community pharmacists and pharmacy support staff, representatives from independent and multiple pharmacy groups, GPs, service commissioners and policy makers ( Griffiths *et al.*, 2022). The practitioner group will be recruited via the extensive networks of the study team and through the national Community Pharmacy Workforce Development Group who has already provided input into the design of this project and confirmed its timeliness and importance. The group will meet up to 6 times during the project. Each of our advisory groups will meet regularly at key milestones throughout the project. The PPIE group will input into the development of patient facing study outputs, dissemination strategy and pathways to impact. Efforts were made to ensure that membership reflects diversity including ethnicity, age, gender, rural/urban living etc. Feedback from both our PPIE and practitioner stakeholder group will inform our relevance criteria as part of the focus of the realist synthesis. The groups will also support with interpretation of the evidence from the papers.

Two members of the PPIE group will also be recruited to the project wider practitioner stakeholder group. This group will meet on project start up as part of a facilitated workshop to develop initial programme theories and will meet regularly through the project to input and give feedback on the study focus, emerging findings, programme theories, proposed interventions, future research and the dissemination strategy and activities, including pathway to impact.

## Stage 2 - Scoping search, locating theory, and developing Initial Programme Theories (IPT)

The purpose of this step is to locate and identify theories that explain how independent prescribing in community pharmacy is supposed to work (or does not work), for whom, when, and why. An initial exploratory search of published and grey literature, in line with realist methodology, will identify, and locate current theories. This stage entails an informal searching strategy that differs from the more formal one discussed in stage 3 ( Pawson *et al.*, 2005). In this stage searching will be aimed at rapidly identifying the range of possible explanatory theories that may be relevant to the programme. The scoping search will commence with broad search terms such as “independent prescribing” and “community pharmacy” via “google scholar” and “one search” engines. Additionally, CLUSTER as a supplementary search framework will be used to allocate key citations and evidence that are directly and indirectly associated to it ( Tsang & Maden, 2021). The CLUSTER framework is designed specifically to enhance complex reviews by incorporating essential contextual information for programme theory development. This framework involves several steps, such as citation searching (tracing references both forward and backward), linking related studies, uncovering concepts associated with the primary research topic, setting boundaries to incorporate relevant perspectives, targeting analogous sources for broader understanding, and evaluating grey literature for additional insights. By expanding the scope of included evidence, the CLUSTER framework adds depth to the review while controlling for potential biases by capturing a diverse range of literature sources and perspectives. This method allows for a rigorous synthesis that supports a comprehensive approach to theory development.

Thus, information gathered at this stage will aid in building initial programme theories to be tested and refined in further stages of this review ( Booth *et al.*, 2018). Finally, the context, intervention, mechanism, outcome (CIMO) framework will be used as a framework to define the review scope and outline keywords ( Booth *et al.*, 2021). This framework was chosen as it matches key realist concepts and definitions. The aim of using this framework is to identify the fundamental elements of the review questions and outline the review’s relevance criteria based on which screening decisions will be made. This will facilitate communicating and continuously refining the scope based on stakeholder’s feedback in a structured manner.

### IPT refinement with expert advisory groups

The first stakeholder groups meetings will be used to inform and acquire stakeholder’s feedback on the scope of the review and help develop the initial programme theory. The groups will consider key issues such as pharmacists’ potential conflicts of commercial interest, separating dispensing and prescribing roles, consultation rooms availability and challenges with access to patient medical records. We will embed discussions relating to these points within each stakeholder group and PPIE meeting; we will also seek to identify and discuss other key issues including unintended consequences of pharmacist-led independent prescribing.

## Stage 3 - Formal search

The realist approach allows us to draw on a wider range of potentially relevant literature that may help us to answer our review questions – e.g., insights gained from the implementation of pharmacist prescribing in other primary and secondary care settings and those from other professions such as nursing ( Pawson *et al.*, 2004), as well as insights gained from the implementation of professional services (using pharmacists’ clinical skills) in community pharmacy as defined by Moullin *et al.* (2013). The purpose of this stage is to identify a relevant body of literature to the defined scope which will be used to further develop and refine the initial programme theory from previous steps. The search strategy to identify published research and grey literature will be developed, piloted, and refined by our information specialist (NR) in collaboration with our team and stakeholder groups. We will include a broad range of evidence, including quantitative and qualitative research, and grey literature such as conference abstracts, preprints, theses, organisational reports, and news items ( Pawson *et al.*, 2005). We anticipate searching databases including MEDLINE, Embase, CINAHL, and International Pharmaceutical Abstracts (IPAB), combining free text and subject heading terms describing community pharmacy settings and independent prescribing activities. We will seek grey literature via relevant organisations and web searches, including pharmacy press such as the Pharmaceutical Journal, Chemists and Druggists, Independent Prescriber, and Pharmacy Magazine from all UK nations. We will also look for relevant international publications in relation to the implementation of prescribing within this setting. We will conduct forward and backward citation tracking to identify additional documents.

Initial scoping searches in Embase and IPAB have identified a range of potentially relevant literature, including 90+ published studies describing or evaluating independent prescribing practices across multiple countries. International literature can provide key insights on other aspects of service delivery including interactions between pharmacists and patients, family carers and the challenges in implementing new service models. We can learn from other countries and will particularly focus on countries with similar healthcare systems including New Zealand and Australia. Only English language studies will be considered. Also, there are no planned date restrictions. We will also draw on learning from previous and current work led by the team to understand the influence of these issues on pharmacists’ prescribing practice. For instance, the centre for pharmacy workforce studies led by one of our team members conducted multiple studies on this topic ( Jacobs *et al.*, 2018). Another member led the PERISCOPE study which looked at community pharmacy led COVID-19 vaccination specifically considering relevant issues around pharmacy consultation rooms ( Maidment *et al.*, 2021).

The iterative realist methodology will allow us to refine and further develop our searches to identify issues such as conflicts of interest due to commercial pressures and access to medical records ( Pawson *et al.*, 2005). Additional searches will be conducted later if additional data are needed for programme theory development. For example, we may devise searches to explore literature that could provide analogous data, including literature describing pharmacists’ independent prescribing in other settings, other professions taking on prescribing roles, and community pharmacists taking on other enhanced roles ( Wong *et al.*, 2015). For each additional search the project team will discuss and determine the relevance criteria. Our information specialist will develop, pilot, and refine any additional searches needed. The screening processes will be as described below for the initial search.

## Stage 4 - Data appraisal and selection

Firstly, articles will be screened by title and abstract and then full-text against inclusion and exclusion criteria ( Pawson *et al.*, 2005). In cases where a definitive selection decision is hard to make, discussions within team members will guide the decision making. A 10% random sample of the citations selected and retrieved by the searches will be reviewed independently by another project team member to check for systematic errors. Any disagreements will be discussed between both team members, with a third member of the team reviewing the citation where required. Finally, full text documents will be included if they have relevant data that can contribute to theory building or testing. Additionally, upon reviewing full texts, assessments of rigour will be made following Wong *et al.* (2015) on a scale from 1 to 5, with 1 indicating low rigor and 5 indicating high rigor. This assessment will focus on each article's explanatory power, specifically evaluating the credibility and trustworthiness of the data presented. Studies will be rated based on factors such as methodological soundness, data validity, transparency in reporting, and reliability of findings. Documents may still be included if judged to be of limited rigor if they provide causal insights for theory refinement, as we will also be making an overarching assessment of rigor at a programme theory level.

Initial inclusion criteria will be as follows: documents that describe or evaluate independent prescribing, or advanced clinical skills in community pharmacy settings; healthcare systems judged comparable to the UK NHS; and published in English. We are initially excluding non-community pharmacists, supplementary and other non-independent prescribing (for example use of Patient Group Direction). Adjustments to the inclusion and exclusion criteria scope will be discussed with project team meeting if needed. No limits will be placed on publication date or study design, as we will seek to consider a range of qualitative, quantitative, and mixed methods studies and grey literature.

## Stage 5 - Data extraction

Data extraction, in terms of selection and coding, will be an iterative process as explained in this section a four-step process ( Dalkin *et al.*, 2021) Full texts of potentially relevant documents will be obtained and uploaded into NVivo. Relevant sections of texts, which have been interpreted as relating to contexts, mechanisms, and their relationships to outcomes, will be extracted, coded, and recorded in NVivo. Coding will be deductive (based on codes created prior to data extraction/analysis and informed by the initial programme theory), inductive (including new codes created to categorise data from studies included), and retroductive (including codes created to infer hidden causal forces for outcomes based on interpretation of data) ( Meyer & Lunnay, 2013). Newly extracted data will be utilised to enhance and refine theory. As each theory is refined, we will revisit the included studies to uncover additional data pertinent to the revised theories. Characteristics of included documents will be extracted and recorded onto an Excel spreadsheet, including bibliographic information and study design, setting and population. A 10% random sample of the extracted data will be cross checked by another team member for quality assurance.

## Stage 6 - Data analysis & Synthesis

A realist logic of analysis will be utilised to make sense of the initial programme theory. We will use a series of questions about the relevance and rigour of content within data sources as part of our process of analysis and synthesis, as set out in Table 1 ( Wong *et al.*, 2013). Analysis will develop context, mechanism, outcome (CMO) configurations embedded within a programme theory which will be further explored through regular team and stakeholder meetings. To interpret how context, mechanisms, and outcomes are interconnected, we will draw on data from all included documents ( Wong *et al.*, 2013); Because individual documents rarely contain every element needed for CMOC configurations, synthesizing information from multiple sources is often required, thus insights about mechanisms found in one source might help explain outcome variations observed in different contexts elsewhere.

**Table 1.** A series of questions used to guide analysis.

Criteria	Questions
Relevance	Are sections of text within this document relevant to programme theory development?
Rigour (judgements about trustworthiness)	Are these data sufficiently trustworthy to warrant making changes to any aspect of the programme theory?
Interpretation of meaning	If the section of text is relevant and trustworthy enough, do its contents provide data that may be interpreted as functioning as context, mechanism, or outcome?
Interpretations and judgements about Context-Mechanism-Outcome-Configurations (CMOCs)	What is the CMOC (partial or complete) for the data that have been interpreted as functioning as context, mechanism, or outcome? Are there further data to inform the particular CMOCs contained within this document or other documents? If so, which other documents? How does this particular CMOC relate to other CMOCs that have already been developed?
Interpretations and judgements about programme theory	How does this particular (full or partial) CMOC relate to the programme theory? Within this same document are there data which informs how the CMOC relates to the programme theory? If not, are there data in other documents? Which ones? Considering this particular CMOC and any supporting data, does the programme theory need to be changed?

In the analysis process described above, interpretive cross case comparisons will be applied to explore and explain the reasons behind the observed outcomes ( Bergeron & Gaboury, 2020). For instance, comparing contexts where independent community pharmacist prescribing has been successfully implemented with contexts when implementation has proved challenging, can help us clarify how the reported findings were shaped by context. When addressing the questions presented in Table 1, we will employ several reasoning techniques to interpret the data where appropriate. Those techniques include juxtaposing, which entails comparing how the independent prescribing setting influenced outcomes in one document to gain insights into outcomes in another. Techniques also include reconciling, entailing the exploration of discrepancies in data from seemingly similar contexts to uncover reasons for those differences. Another technique is adjudication, which refers to resolving conflicting data by assessing the methodological rigor of the data collection methods. Finally, consolidation will be employed to explain variations in outcomes across different contexts by identifying the underlying reasons ( Gilmore *et al.*, 2019; Mukumbang, 2023).

Our analyses will aim to identify the most effective ways to implement independent community pharmacist prescribing and the strategies we might be able to use to change existing contexts in such a way that ‘key’ mechanisms are triggered to produce desired outcomes (e.g., safe, and effective prescribing). We plan (given the data available) to understand how pharmacist independent prescribing could work in different circumstances such as, different communities including ethnic minority populations and other potentially marginalised groups and different areas e.g., rural vs urban, deprived vs affluent.

### Development of recommendations

The overarching goal for this research is to improve our understanding of how independent prescribing in community pharmacy works, why it works (or not), and in what circumstances, so we can recommend strategies to maximise widespread implementation and utilisation to meet the needs of the NHS and patients and take a step towards achieving ICS goals ( Charles *et al.*, 2018). The programme theory developed and refined in our review will be used to develop recommendations to support the widescale implementation of independent prescribing in community pharmacy ( Wong *et al.*, 2015). Output will be generated as follows.

**Outputs for policy makers/clinicians:** our research will provide evidence-based recommendations for policy makers, pharmacy owners/employers, commissioners, clinicians, and higher education institutes, and may have implications for how community pharmacy prescribing is integrated into primary care systems. This audience is key to implementing recommendations made in our review, hence it is crucial to involve key individuals in our stakeholder group and work with them throughout the review. We will work with our stakeholder group to develop briefing materials that are tailored to different audiences. We will also target pharmacy trade publications (e.g., Pharmaceutical Journal, Chemist and Druggist) to ensure dissemination to a wider audience of pharmacists and contractors.

**Outputs for academics/researchers**: our findings will likely be relevant for academics planning research into community pharmacy based prescribing interventions and workforce development. We will also identify areas where current evidence is lacking, providing recommendations for future research in this field. Outputs will include a formal project report, academic publications, conference presentations and dissemination through the extensive networks of the team and stakeholder group.

**Outputs for service users:** we will develop information and patient facing guidance on how prescribing by community pharmacists can be best delivered for certain groups of people. We will publish findings on a project website and through patient and third sector groups. The PPIE group will support the development of the outputs and dissemination strategy.

### Equality, diversity, and inclusivity

Equality, diversity and inclusivity (EDI) is embedded throughout this project following NIHR strategy ( NIHR, 2022). We have and will continue further recruitment to both the PPIE and practitioner groups. Our PPIE group is made up of individuals from diverse backgrounds. This will ensure that the voices of underserved groups, including people of different ethnic groups, gender, sexual orientation, people with disability and long-term conditions, people from rural and urban locations, and people living in areas of low socioeconomic status. We will also consider outcomes relating to EDI in our review, where evidence around who does (or does not) access community pharmacy services is identified. The strong PPIE presence in the research team, practitioner group and PPIE group will ensure that the study is conducted with and by service users.

## Write up and dissemination of review findings and recommendations

We will adopt an integrated approach to dissemination based on NIHR Guidance “How to disseminate your research” (2019a); this will include dissemination locally, nationally and internationally to achieve impact. Our dissemination strategy will leverage the participatory approach that we have employed during the development of this research proposal by involving stakeholders and will continue to utilize throughout the planned research. Our review will be of interest to a diverse group of stakeholders, such as pharmacists, health care professionals, policy makers, patients, and researchers, who will find value in the findings and recommendations. We will adopt different strategies informed by our stakeholder’s expertise to identify the primary audiences for dissemination. Additionally, we will create customised material pertinent to each specific audience.

## Ethics and consent

Ethical approval is not required, because primary data will not be collected.

## Data Availability

No data are associated with this article. Underlying data: No underlying data associated with this article. Extended data: No extended data are associated with this article.

## Software availability

NVivo is proprietary software, available at https://lumivero.com/products/nvivo/. Free alternatives such as RQDA (R package for qualitative data analysis) can perform similar functions. RDQA is available at https://rqda.r-forge.r-project.org/.
