# Supplementary material for: INdependenT prEscribinG in community phaRmAcy; whaT works for whom, why and in what circumstancEs (INTEGRATE): Realist review study protocol
Source: NIHR Open Res. 2024 Nov 18;4:72. [Version 1] doi: 10.3310/nihropenres.13766.1 (PMC11808844; doi:10.3310/nihropenres.13766.1)
Supplement: Peer review history

# Reviewer report: Reviewer response for version 1

**DOI:** 10.3310/nihropenres.14953.r34013

**Published:** 2025-01-06

**Authors:** Arthur Piraux

**Affiliations:** 1 Univ Angers, Angers, France

This study, entitled INTEGRATE, is part of a large-scale study focusing on independent prescribing by community pharmacists. The protocol for this realistic study includes six successive and complementary stages, and the main objective is to understand how independent prescribing works in community pharmacy, and the context in which it is implemented.

 This study protocol is clear and precise, it contains all the elements essential to a good understanding of the study. Each of the six steps has been explained and justified. It is important to emphasize the robustness of the methodology, backed up by multiple references, as well as the involvement of patient stakeholders from the outset. Moreover, the protocol is also available online on the PROSPERO platform.

 Can the authors predict how they would like to select the various articles? Is specialized software (e.g. COVIDENCE) envisaged? Or more manual processing (e.g. Microsoft Excel)?

 I have no further comments in view of the quality of this very comprehensive article. The methodology employed augurs well for the future validity of the results, which we eagerly await.

Is the study design appropriate for the research question?

Yes

Is the rationale for, and objectives of, the study clearly described?

Yes

Are sufficient details of the methods provided to allow replication by others?

Yes

Are the datasets clearly presented in a useable and accessible format?

Not applicable

Reviewer Expertise:

I participated in a scoping review dealing with independent prescribing by the community pharmacist. To a lesser extent, I am working with organizations representing the profession to roll out this prescribing method, which is still underdeveloped in France.

I confirm that I have read this submission and believe that I have an appropriate level of expertise to confirm that it is of an acceptable scientific standard.

# Reviewer report: Reviewer response for version 1

**DOI:** 10.3310/nihropenres.14953.r34014

**Published:** 2025-01-02

**Authors:** Veronica Vernon

**Affiliations:** 1 Butler University College of Pharmacy and Health Sciences, Indianapolis, USA

Thank you for undertaking this important work. I foresee this study having an impact not just in the United Kingdom, but also internationally. Implementation of pharmacist prescribing is an area of interest in other countries where uptake has not been as widespread as anticipated. I do think it would be useful, but potentially not realistic, to try to obtain data from as many pharmacies in the UK as possible to determine why they are not implementing pharmacist prescribing services. 

 Introduction

 -Is there any data to show how many pharmacists have completed the independent prescribing training? How many pharmacists and pharmacies implemented the Pharmacy First program? I would be curious to know the “current state.”

 Methods

 -Stage 1: This is an incredibly important piece. This is wonderful!

 I appreciate the mention of EDI in stage 6. I wonder if you need to mention some of the EDI considerations in stage 1, as your recruitment of advisory group members is intentional

Is the study design appropriate for the research question?

Yes

Is the rationale for, and objectives of, the study clearly described?

Yes

Are sufficient details of the methods provided to allow replication by others?

Yes

Are the datasets clearly presented in a useable and accessible format?

Not applicable

Reviewer Expertise:

Pharmacy law and regulations, pharmacist prescribing, pharmacist-prescribed contraception

I confirm that I have read this submission and believe that I have an appropriate level of expertise to confirm that it is of an acceptable scientific standard.

# Reviewer report: Reviewer response for version 1

**DOI:** 10.3310/nihropenres.14953.r33817

**Published:** 2024-12-24

**Authors:** Mohammed S Alharthi

**Affiliations:** 1 College of Pharmacy, Clinical Pharmacy Department., Taif University, Taif, Makkah Province, Saudi Arabia

Summary of the Article

 The study protocol describes a realist review aiming to explore how independent prescribing in community pharmacies works, for whom it works, under what circumstances, and why. The study responds to workforce challenges in the NHS and aligns with the NHS Long-Term Plan, which emphasizes the potential of community pharmacists to provide patient-centered care through prescribing. The six-stage realist methodology will investigate the contexts, mechanisms, and outcomes (CMO) that influence independent prescribing in community pharmacy settings. Key features include stakeholder engagement with patients and practitioners to co-develop programme theories and recommendations for policymakers, practitioners, and researchers. The study is timely, given the upcoming policy change that will allow pharmacists to qualify as independent prescribers upon graduation starting in 2026.

 Evaluation of Key Questions

 1. Is the rationale for, and objectives of, the study clearly described?

 The study provides a clear and comprehensive rationale by addressing current pressures on the NHS workforce and the potential role of pharmacists as independent prescribers. The objectives align with the overarching research question and are subdivided into specific sub-questions, ensuring clarity and focus.

 2. Is the study design appropriate for the research question?

 The use of a realist review is well-justified given the complexity of the intervention being studied. Realist methodology is appropriate for understanding how and why independent prescribing works in different contexts and for different populations. The inclusion of stakeholder engagement at multiple stages adds depth and practical relevance to the study design.

 3. Are sufficient details of the methods provided to allow replication by others?

 While the methodology is generally well-structured, certain aspects need more detail to ensure replicability. The protocol mentions broad inclusion and exclusion criteria, such as 'healthcare systems comparable to the NHS,' but does not specify how these systems will be identified or what metrics will be used for comparison. The search strategy is described in general terms, but specific search terms, databases, and the approach to grey literature retrieval are not detailed. The coding process in NVivo is mentioned but lacks sufficient explanation of how the coding framework will evolve and how inter-coder reliability will be ensured.

 4. Are the datasets clearly presented in a usable and accessible format?

 Since this is a study protocol, there are no datasets to present. The description of how data will be extracted and synthesized is clear and appropriate for the planned realist review.

 Recommendations for Improvement

 First, clarify operational definitions and criteria. This includes providing detailed inclusion and exclusion criteria, with specific parameters for 'comparable healthcare systems,' and defining terms such as 'contexts,' 'mechanisms,' and 'outcomes' in practical terms to guide data extraction and analysis. Second, enhance search strategy transparency by including specific keywords, databases (e.g., MEDLINE, Embase, CINAHL), and the process for identifying grey literature. The application of the CLUSTER framework should also be explained in more detail. Third, provide more detail on coding and analysis. Elaborate on the coding process, including examples of deductive, inductive, and retrodictive codes, and clarify steps for ensuring coding consistency, such as inter-coder reliability checks. Finally, systematically integrate stakeholder feedback. Detail how feedback from PPIE and practitioner stakeholders will be recorded, analyzed, and incorporated into the refinement of programme theories.

 Points to Address for Scientific Soundness

 To ensure scientific soundness, the protocol should address the following points. First, provide detailed inclusion and exclusion criteria to ensure clear and reproducible criteria for literature screening, including the definition of 'comparable healthcare systems.' Second, develop a comprehensive search strategy that includes a full description of the search terms, databases, and grey literature sources to be used. Third, expand on the coding process by offering a detailed explanation of how coding will be conducted and validated. Finally, clearly outline the process for incorporating stakeholder feedback into programme theories and recommendations.

 Conclusion

 The protocol is a timely and well-conceived study that addresses a critical issue in healthcare. While the rationale, objectives, and overall methodology are robust, the protocol would benefit from additional detail in areas such as inclusion/exclusion criteria, search strategy, and coding processes to enhance replicability and scientific rigor. Addressing these areas will strengthen the study’s potential to produce actionable and impactful findings.

Is the study design appropriate for the research question?

Yes

Is the rationale for, and objectives of, the study clearly described?

Yes

Are sufficient details of the methods provided to allow replication by others?

Partly

Are the datasets clearly presented in a useable and accessible format?

Not applicable

Reviewer Expertise:

My area of interest is medication optimization, health service research, mixed methods, consensus methods, process evaluation, quantitative research, qualitative research, systematic reviews, scoping reviews, and medication deprescribing.

I confirm that I have read this submission and believe that I have an appropriate level of expertise to confirm that it is of an acceptable scientific standard.
